# Effects of isoflurane and xylazine on inducing cerebral ischemia by the model of middle cerebral artery occlusion in mice

**DOI:** 10.1186/s42826-023-00163-6

**Published:** 2023-06-01

**Authors:** Jinyoung Won, Zeeshan Ahmad Khan, Yonggeun Hong

**Affiliations:** 1 Department of Rehabilitation Science, Graduate School of Inje University, 197 Inje-ro, Gimhae, Gyeong-nam 50834 Republic of Korea; 2 Research Center for Aged-life Redesign (RCAR), Inje University, Gimhae, Republic of Korea; 3 Biohealth Products Research Center (BPRC), Inje University, Gimhae, Republic of Korea; 4 Department of Physical Therapy, College of Healthcare Medical Science and Engineering, Inje University, Gimhae, Republic of Korea

**Keywords:** Anesthesia, Middle cerebral artery occlusion, Ischemic stroke, Isoflurane, Xylazine

## Abstract

Preclinical ischemic stroke studies extensively utilize the intraluminal suture method of middle cerebral artery occlusion (MCAo). General anesthesia administration is an essential step for MCAo, but anesthetic agents can lead to adverse effects causing death and making a considerable impact on inducing cerebral ischemia. The purpose of this study was to comparatively assess the effect of isoflurane and xylazine on transient cerebral ischemia in a mouse model of MCAo. Twenty animals were randomly divided into four groups: sham group (no MCAo), control group (MCAo under isoflurane, no agent till reperfusion), isoflurane group (MCAo under isoflurane continued till reperfusion), xylazine group (MCAo under isoflurane, and administration of xylazine till reperfusion). The survival rate, brain infarct volume, and neurologic deficits were studied to assess the effect of isoflurane and xylazine on the stroke model. Our results showed that the body weight showed statistically significant change before and 24 h after surgery in the control and Isoflurane groups, but no difference in the Xylazine group. Also, the survival rate, brain infarct volume, and neurologic deficits were slightly reduced in the isoflurane group at 24 h after reperfusion injury. However, the xylazine and control groups showed similar BIV and neurologic deficits. Interestingly, a high survival rate was observed in the xylazine group. Our results indicate that the modified method of inhalation anesthetics combined with xylazine can reduce the risk of mortality and develop a reproducible MCAo model with predictable brain ischemia. In addition, extended isoflurane anesthesia after MCAo is associated with the risk of mortality.

## Background

Cerebral ischemia (CI) is a condition that causes accelerated brain aging, motor function impairment, cognitive decline, and mortality. Due to the irreversible neuronal injury caused by ischemic stroke, extensive preclinical stroke research has been conducted [1]. The middle cerebral artery (MCA) region is where around 88% of ischemic strokes occur [2]. Several models of ischemic stroke, including intraluminal MCA occlusion (MCAo), photothrombosis, and endothelin-1, are currently utilized for animal stroke studies [3]. Among these models, MCAo by intraluminal monofilament insertion is the most frequently used because it closely mirrors the general pattern of the human ischemic brain and can be used for both transient and permanent focal CI [4]. However, the MCAo model has limitations in producing reproducible CI due to surgical manipulation under anesthesia.

General anesthesia is used in in vivo experiments to induce immobility and unconsciousness and as an analgesic [5]. There are two major types of general anesthetic agents: injectable and inhalational anesthetics. Injectable anesthetics, including pentobarbital, ketamine, and propofol rapidly induce loss of consciousness with a small dose [6, 7]. However, injectable anesthetics cause medullary paralysis and sudden cardiac arrest due to unpredictable anesthetic depth [8]. Inhalational anesthetics, like ether and isoflurane, are used in experimental surgical procedures regardless of the species [9]. Isoflurane has several benefits, including fast recovery, prompt elimination, low hepatotoxicity, and low mortality [10]. However, exposure to high concentrations of inhalational anesthetics can cause deficits, including apnea, hypoxia, and unconsciousness [11].

Low mortality and reproducible brain infarct volume (BIV) in rodent stroke models are affected by various factors, including surgical technique, environmental variation, and prolonged duration of surgery under general anesthesia [12, 13]. Particularly, the choice and time of anesthesia and analgesia can significantly affect stroke model outcomes [14]. The objective of the present study is to explore an appropriate method and timing of anesthesia to avoid potential side effects that could result in unexpected complications in the MCAo model. We conducted a comparative study to evaluate the effects of isoflurane and xylazine on MCAo-induced ischemic injury in mice to achieve this goal.

## Materials and methods

### Animals

The animal study was approved by the Institutional Animal Care and Use Committee (IACUC) at Inje University (approval no. 2015-11, 2018-005) and conducted following the Inje University Animal Care guidelines and the Korean Department of Agriculture. All animals were maintained under a 12 h dark/light cycle with free access to laboratory chow and drinking water. C57BL/6 male mice weighing 20–25 g (8-week-old) were used in this experiment.

### Experimental groups

**Fig. 1:**
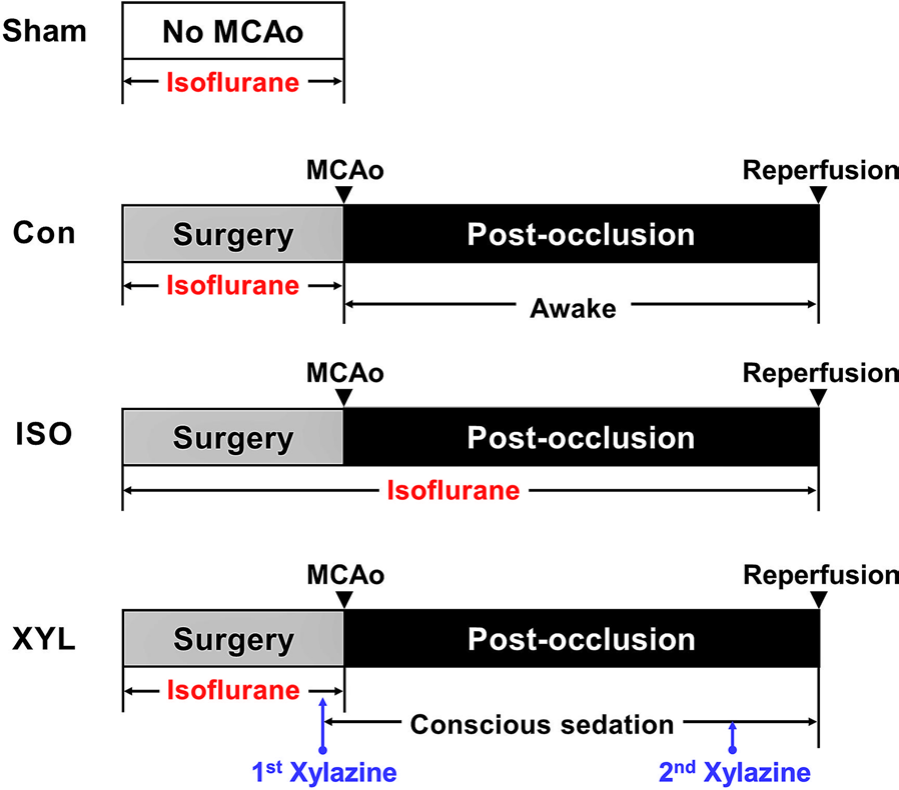
Schematic presentation of the experimental design. Animals were subjected to transient focal ischemia for 60 min after MCAo. Animals were randomly divided into four groups: sham group (no MCAo), control group (Con, MCAo under Isoflurane; no agent till reperfusion), isoflurane group (MCAo under Isoflurane, 1.5% isoflurane anesthesia continued till reperfusion), xylazine group (MCAo under Isoflurane, intramuscular xylazine injection (20 mg/kg) 10 min before artery occlusion, and 30 min post-occlusion). 5 (sham), 14 (control), 25 (ISO), 25 (Xyl) animals were used in this study

Sixty-nine animals were used in this study. Animals were randomly divided into four groups: sham group (no MCAo, n = 5), control group (MCAo under Isoflurane; no agent, n = 14), isoflurane group (MCAo under Isoflurane, 1.5% isoflurane anesthesia continued till reperfusion, n = 25), xylazine group (MCAo under Isoflurane, intramuscular xylazine injection (20 mg/kg) 10 min before artery occlusion, and 30 min post-occlusion, n = 25) (Fig. 1).

### Focal ischemic-reperfusion stroke model

The experimental stroke model was performed according to the modified Longa model [15]. The MCAo was induced by an intraluminal suture in the MCA. The duration of surgical procedures did not exceed 20 min, and the operation time from the induction of anesthesia to artery occlusion was limited to 30 min. The right common carotid artery (CCA) was carefully separated from the vagus nerve and ligated temporarily. The CCA was bifurcated into the external carotid artery (ECA) and internal carotid artery. A monofilament (6.0; silicon-coated tip, 0.22–0.23 mm; Doccol Corporation, CA, USA) was used to occlude blood flow. The suture was inserted 9 to 11 mm into the ECA to block the origin of the right MCA. Reperfusion by removal of monofilament was performed after 60 min occlusion. The body temperature of the mice was maintained at 37 ± 1 °C during occlusion and after reperfusion. Sham groups underwent the same anesthesia and surgical procedures as MCAo groups, except the intraluminal filament was not advanced to the origin of the MCA.

### Induction and maintenance of anesthesia

Animals were anesthetized with isoflurane in a mixture of 30% O_2_ and 70% N_2_O using the anesthesia system (Harvard Apparatus Inc., Holliston, MA, USA). Induction of anesthesia was mediated by 3% isoflurane in a sealed chamber. After the isoflurane induction, anesthesia maintenance was achieved by 1.5% isoflurane via facemask during MCAo surgery, taking about 20 min.

### Evaluation of neurological deficit

Neurological deficits were scored in a blind manner using a neurological disability status score after 72 h of reperfusion, which grades neurological deficiencies from 0 (normal) to 10 (most severe injury/death) (Table 1). The precise grade was based on neurobehavioral alterations that were divided into two phases [16].

**Table 1:** Neurological disability status scale (NDSS)

Degree of deficit	Neurobehavioral alterations
0	None
2	Hypomobility (slight) Passivity
4	Hypomobility (moderate) Flattened posture Lateralized posture Hunched back Ataxic gait Piloerection Decreased body tone Decreased muscular strength Motor incoordination (slight)
6	Circling Tremor/twitches/convulsions Forelimb flexion Motor incoordination (moderate)
8	Hypomobility (severe) Motor incoordination (severe) Respiratory distress
10	Death (due to MCAo)

### Quantification of infarct volume

2,3,5-triphenyl tetrazolium chloride (TTC) was used to determine the BIV. Animals were subjected to MCAo, euthanized at 72 h, and perfused with 0.01 M phosphate-buffered saline (pH 7.4) immediately following sacrifice in order to minimize autolysis which begins in the absence of oxygen following death. Using a mouse brain slicer, the brain was cut into 1 mm thick slices for analysis of the infarct area and volume (Mouse Brain Matrix; ASI Instruments, Warren, MI, USA). Brain sections were immersed for 10 min in 2% TTC (Sigma-Aldrich, St. Louis, MO, USA) at 37 °C. The infarct area (mm^2^) was measured using the set scale function of Image J software (NIH, Bethesda, MD, USA). The brain infarct area, excluding brain tissue edema, was based on the contralateral hemisphere area. The sum of the measured infarct area is used to calculate the total BIV (mm^3^) according to modified trapezoidal and Simpson’s rule [17].

### Statistical analysis

Data were collected from repeated experiments and are presented as means ± standard deviation (SD). Statistically significant differences between groups were assessed using one-way ANOVA with the post hoc Tukey’s test. Statistical significance was set at *p* < 0.05. All data were analyzed using SPSS software (IBM, New York, NY, USA).

### Experimental procedure and impact of MCAo-induced CI on physiological responses

**Fig. 2:**
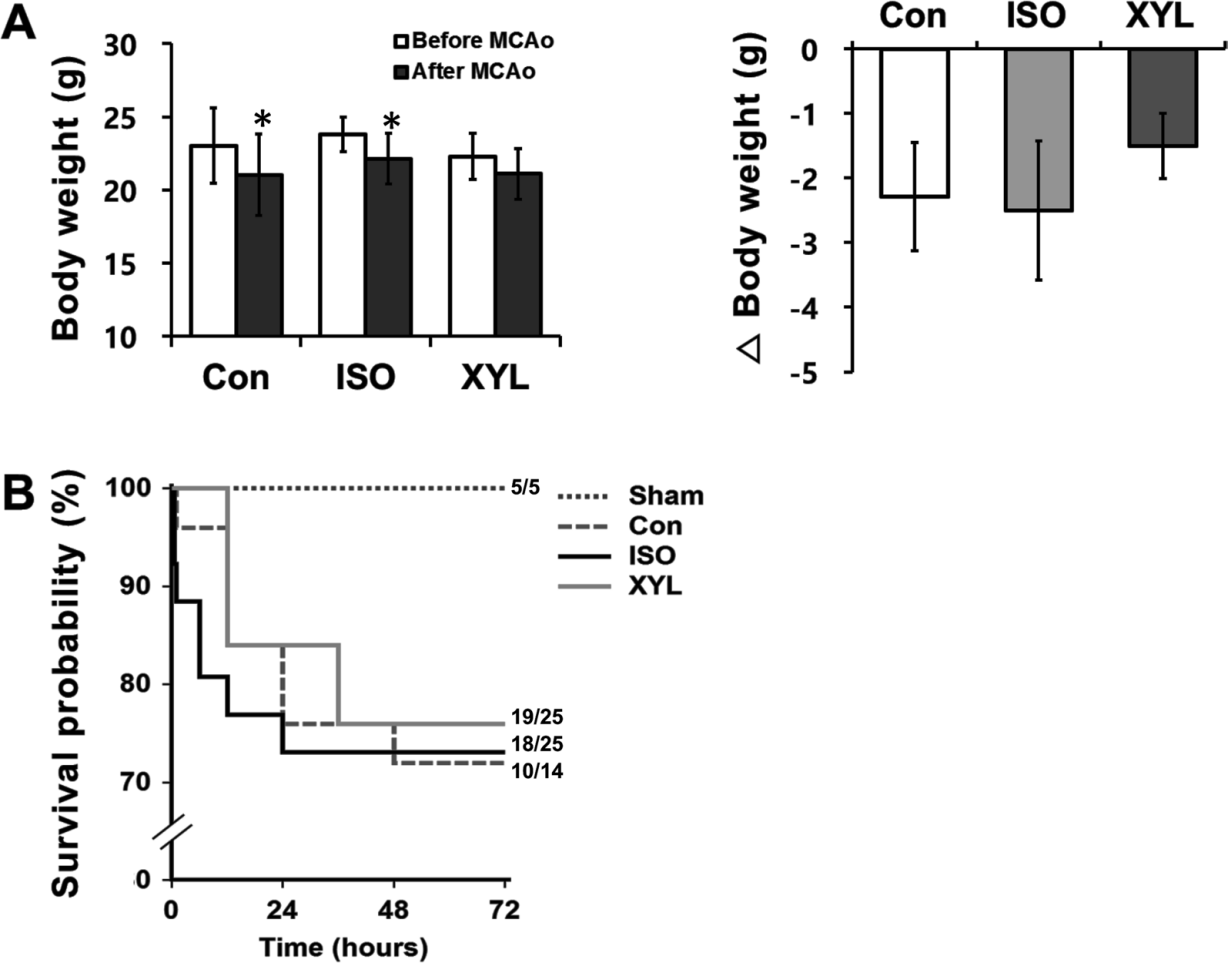
Effect of MCAo-induced cerebral ischemia and prolonged anesthesia on physiological activity and survival rate. **A** Alterations from baseline body weight to 24 h after MCAo. Body weight was measured before the MCAo and 72 h post-reperfusion. Data are presented as mean ± S.D. **B** Survival rate is defined as the percentage of surviving animals from induction of ischemia-reperfusion until ischemic brain injury assessment. Survival data were expressed by the Kaplan**–**Meier curve, and compared using the log-rank test. 5 out of 5, 10 out of 14, 18 out of 25, and 19 out of 25 animals survived in sham, control, ISO, and Xyl groups, respectively. Mortality data were recorded for 72 h to conduct a survival analysis. 5 (sham), 14 (control), 25 (ISO), and 25 (Xyl) animals were used in this study. Data are presented as mean ± S.D *p*-value was considered significant at *p < 0.005

The MCAo surgery was conducted on animals under general anesthesia using isoflurane. Following MCAo induction, the animals were divided into sham, control, isoflurane, and xylazine groups at random (Fig. 1). Baseline weight was measured immediately before MCAo, and the body weight was monitored every 24 h post-reperfusion for 72 h. Animals in control and isoflurane showed a significant decline in body weight following MCAo as compared with baseline weight. However, there was a statistically significant difference between the control and xylazine group (panel A in Fig. 2). These alterations indicate that physiological change might be associated with CI induced by MCAo.

### The effect of prolonged anesthesia on survival rate in MCAo

Survival data were presented in the Kaplan-Meier curve and data analyses were performed using the log-rank test (panel B in Fig. 2). No mortality was observed in the sham group, indicating a lack of acute toxicity of anesthetics. A significant change in the survival curve was observed within 24 h after reperfusion in the control and isoflurane groups. Sudden death during post-occlusion mainly occurred in the isoflurane group, and a high risk of mortality at 1 h after reperfusion was observed in the control group. After 72 h, the control group showed the lowest survival rate (~ 71%) followed by the isoflurane group (~ 73%). On the other hand, the xylazine group showed a high survival rate of ~ 77% at 72 h. The sedative and analgesic effects of xylazine might cause a positive result in the survival rate [18, 19].

### A comparison of ischemic outcome between awake and anesthetized states during transient CI by MCAo

**Fig. 3:**
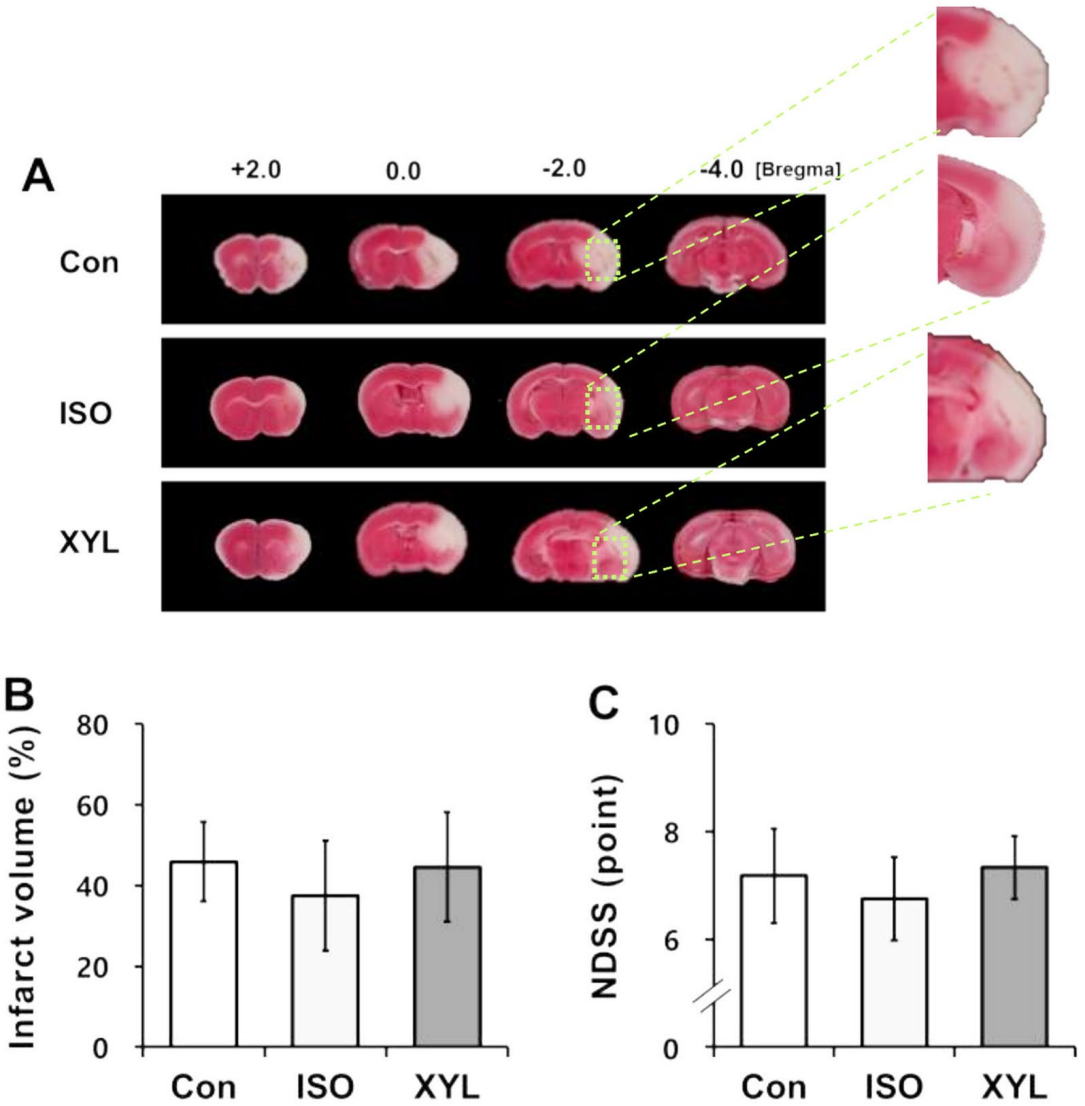
Comparison of ischemic brain injury in MCAo mice. **A** Representative images of TTC-stained brain slices from bregma + 4.0 mm to bregma − 4.0 mm. Ischemic injury induced by MCAo was measured at 72 h post-reperfusion. **B** Quantitative analysis of infarct volume. Total infarct volume was measured using a TTC image and presented as a percentage of contralateral hemisphere volume. **C** Quantitative assessment of neurologic deficits. Neurobehavioral alteration resulting from ischemic injury was assessed by NDSS at 72 h after reperfusion. Data are presented as mean ± S.D. 5 (sham), 14 (control), 25 (ISO), and 25 (Xyl) animals were used in this study

We compared the BIV and behavior outcome to determine the effect of different anesthesia during MCAo. The BIV was visualized by TTC staining (panel A in Fig. 3). The BIV (panel B in Fig. 3) and neurologic deficits were reduced in the isoflurane group compared with the control and the xylazine group (panel C in Fig. 3). However, there was no statistically significant difference between the groups. The xylazine group showed that ischemic outcomes including BIV and neurological dysfunction were similar to those in the control group. Thus, we suggest that the modified MCAo method under conscious conditions administered xylazine could reduce the risk of mortality and enhance the high reproducibility of the MCAo model.

### Implications of the present research

We showed that animals subjected to MCAo have shown a decline in weight. Bodyweight is used as a potential supplementary parameter for predicting BIV and neurological impairment in preclinical stroke research [20, 21]. Previous studies demonstrated a significant decrease in body weight by approximately 10% at 24 h after the onset of MCAo [22]. In contrast, when exposed to isoflurane anesthesia, sham animals showed no significant changes in body weight over time. However, animals in the control and isoflurane groups showed a significant decrease in body weight following MCAo compared to their baseline weight. Interestingly, there was a significant difference in body weight between the control and xylazine groups. These findings suggest that physiological responses are associated with brain injury caused by MCAo but not affected by isoflurane anesthesia.

We observed that the xylazine group achieved a higher survival rate than that of other groups, while the sham group exhibited no mortality. Although convulsive seizure and hemorrhage did not occur, unexpected death with cardiac arrest was observed in the prolonged isoflurane anesthesia group. The results of the present study are consistent with those found in previous studies which showed that anesthetics can affect the cardiovascular system, including the heart, blood vessels, and arterial blood pressure [23]. Animals exposed to prolonged isoflurane during the occlusion period showed a significant reduction in mean arterial pressure by approximately 20–25 mmHg, which may explain the relationship between anesthesia-related hypotension and cardiac arrest [24]. Also, survival results indicate that isoflurane anesthesia might be associated with an increased risk of mortality after MCAo probably because isoflurane has little to no analgesic potency [18]. Therefore, with this study, we suggest that long-term anesthesia with isoflurane during MCAo can decrease the survival of animals.

Finally, the BIV and neurologic deficits were assessed to investigate the effect of anesthesia on CI. Although the survival rate was increased in the xylazine group compared to the other groups, the extent of brain damage was highly sensitive to transient MCAo and reperfusion injury. The xylazine group presented slightly more infarction and neurologic dysfunction compared with those observed in the isoflurane group. Vascular endothelial growth factor (VEGF) is a major regulator of normal and pathologic blood vessel growth. However, VEGF also has the unique property of inducing vascular leakage [25, 26]. Matrix metalloproteinase-9 (MMP-9) is an enzymatic protein that degrades the extracellular matrix and may cause degradation of the blood–brain barrier (BBB) after MCAo [27]. The MMP-9 might be activated by VEGF [28]. Thus, MMP-9 activation may cause both breakdowns of the BBB and intracranial hemorrhage after MCAo. Anesthetic treatment in ischemia-reperfusion models results in the down-regulation of MMP-9 and VEGF expression [22, 29], thereby reducing the BIV.

### Limitations and future prospective

A limitation of this study is the lack of postoperative analgesia administration. We chose not to administer analgesics due to the dual analgesic and anesthetic effects of xylazine used with isoflurane [18]. The inclusion of postoperative analgesia could have influenced the study outcome. Further research is recommended to compare the effects of isoflurane and xylazine on transient CI in a mouse model of MCAo, while considering postoperative analgesics. This would provide a more comprehensive evaluation of analgesic impact on study outcomes, while prioritizing animal welfare. Further analysis of VEGF and MMP-9 mRNA and protein is needed to confirm the impact of modified anesthesia on intracranial hemorrhage after MCAo and determine the efficacy of isoflurane in reducing cerebral ischemic injury. Additional research is necessary to draw definitive conclusions about the effectiveness of isoflurane.

## Conclusions

We demonstrated that minimized use of isoflurane improves the survival rate in a mouse model of MCAo. The modified method of isoflurane combined with xylazine for conscious sedation could reduce the risk of mortality and provide a reproducible MCAo model. In conclusion, we suggest that the experimental animal models requiring general anesthesia should avoid prolonged periods of anesthetic exposure.

## Data Availability

[REAGENTS/TOOLS/MATERIALS] generated in this study are available from the corresponding author upon request.

## Abbreviations

BBB: Blood–brain barrier
BIV: Brain infarct volume
CI: Cerebral ischemia
CCA: Common carotid artery
MMP-9: Matrix metalloproteinase-9
MCAo: Middle cerebral artery occlusion
NDSS: Neurological disability status score
TTC: 2,3,5-Triphenyl tetrazolium chloride
VEGF: Vascular endothelial growth factor
